# Insight into the exemplary structural, elastic, electronic and optical nature of GaBeCl_3_ and InBeCl_3_: a DFT study

**DOI:** 10.1039/d2ra00943a

**Published:** 2022-03-15

**Authors:** Saima Ahmad Shah, Mudasser Husain, Nasir Rahman, Mohammad Sohail, Rajwali Khan, Abid Ali Khan, Asad Ullah, Shaimaa A. M. Abdelmohsen, Ashraf M. M. Abdelbacki, Ahmed M. El-Sabrout, Hosam O. Elansary, Aurangzeb Khan

**Affiliations:** Department of Physics, Shaheed Benazir Bhutto Women University Peshawar Pakistan; Department of Physics, Abdul Wali Khan University Mardan Pakistan nasir@ulm.edu.pk akhan@awkum.edu.pk; Department of Physics, University of Lakki Marwat 28420 Lakki Marwat Khyber Pukhtunkhwa Pakistan; Department of Chemical Sciences, University of Lakki Marwat 28420 Lakki Marwat Khyber Pukhtunkhwa Pakistan; Department of Physics, College of Science, Princess Nourah Bint Abdulrahman University P. O. Box 84428 Riyadh 11681 Saudi Arabia; Deanship of Skills Development, King Saud University Riyadh 11451 Saudi Arabia; Applied Studies and Community Service College, King Saud University Riyadh 11451 Saudi Arabia; Department of Applied Entomology and Zoology, Faculty of Agriculture (EL-Shatby), Alexandria University Alexandria 21545 Egypt; Plant Production Department, College of Food and Agriculture Sciences, King Saud University Riyadh 11451 Saudi Arabia; University of Lakki Marwat 28420 Lakki Marwat Khyber Pukhtunkhwa Pakistan

## Abstract

In the scheme of density functional theory (DFT), Structural, elastic, electronic, and optical properties calculations of GaBeCl_3_ and InBeCl_3_ are carried out using Tran–Blaha modified Becke–Johnson exchange potential approximation (TB-mBJ) installed in Wein2k software. Structurally the compounds of interest are found to be stable. Both compounds possess elastic stability, anisotropy, and ductility determined by the elastic studies. The electronic-band structure analysis shows the semiconductor nature of GaBeCl_3_ and InBeCl_3_ compounds with indirect band gaps of ∼3.08 eV for GaBeCl_3_ and ∼2.04 eV for InBeCl_3_ along with the symmetrical points from (*X*–*Γ*). The calculated total density of states (TDOS) and partial density of states (PDOS) of these compounds reveal that for the GaBeCl_3_ compound, the contribution of Ga (4p) and Cl (3p) orbital states in the valence, as well as the conduction band, is dominant. While for InBeCl_3_, the contribution of Cl (3p) states as well as In (5s) is large in the valence band and in that of Cl (3p-states) states in the conduction band. The type of chemical bonding is found to be ionic in both compounds. The optical properties *i.e.*, the real (*ε*_1_(*ω*)) and imaginary (*ε*_2_(*ω*)) parts of dielectric function, refractive index *n*(*ω*), optical reflectivity *R*(*ω*), optical conductivity *σ*(*ω*), absorption coefficient *α*(*ω*), energy loss *L*(*ω*) and electron extinction coefficient *k*(*ω*) are also discussed in terms of optical spectra. It is reported that *n*(*ω*) and *k*(*ω*) exhibit the same characteristics as *ε*_1_(*ω*) and *ε*_2_(*ω*) respectively. Efficient application of these materials can be seen in semiconducting industries and many modern electronic devices.

## Abstract 2

In the scheme of density functional theory (DFT), Structural, elastic, electronic, and optical properties calculations of GaBeCl_3_ and InBeCl_3_ are carried out using Tran–Blaha modified Becke–Johnson exchange potential approximation (TB-mBJ) installed in Wein2k software.

## Graphical abstract

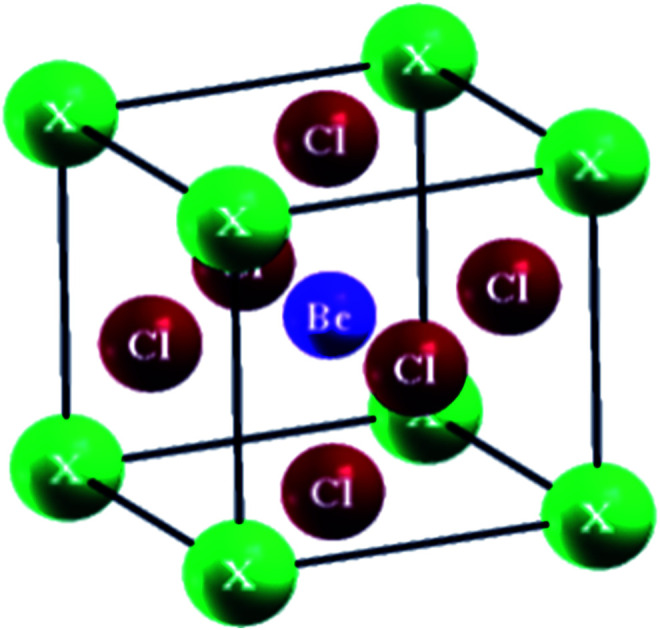

## Introduction

It is well known that perovskite compounds show significant diversity in mechanical,^1,2^ electrical,^3,4^ magnetic,^5^ optical,^6,7^ and transport properties^8–12^*i.e.*, magnetism, ferroelectricity, multiferroicity, superconductivity, piezoelectricity, colossal magnetoresistance, *etc.*^8–16^ And due to these properties, perovskites are the key materials for several technological applications including optoelectronic, photonic, and spintronic appliances *i.e.*, a promising candidate for radiation detection, photovoltaic absorbance and a laser medium.^17–21^ Literally, the flexible crystal structure and elements of which a perovskite is composed of are responsible for some of the characteristics of these perovskites.^22–24^ An ideal perovskite having a cubic crystal structure has the general formula ABX_3_, in which, “A” (occupying the corners) and “B” (occupying the center) are metallic cations while X (occupying the face centers) is an oxide or halide *i.e.*, Cl, Br, I and F. The cubic perovskites are considered as the most stable. It is observed experimentally and theoretically by many scientists that such type of perovskites exhibit the cubic structure possessing the space group *pm̄*3*m* (#221).^25,26^

Metal halide perovskites have several similar phenomena, which exist in the oxide perovskites. Although many perovskites have been found experimentally, the huge number of possible combinations of three chemical elements, the perovskites family may be vast, and possibly many systems are still waiting for researcher discovery.

This work is based on the study of the structural, elastic, electronic, and optical properties of GaBeCl_3_ and InBeCl_3_ perovskites. So far, there has been no experimental or theoretical study on these perovskite materials. Therefore, the work of this paper can be used for reference in the future.

### Computational methodology

The following investigation is performed using TB-mBJ in the framework of DFT, implemented in the WIE2K code.^27^ The TB-mBJ potential is used to investigate the elastic, electronic, and optical properties. Structural properties are computed by fitting the Birch–Murnaghan equation of state (EOS), in which the unit cell volume *versus* unit cell energy is fitted to attain the ground state parameters. The value of RMT is chosen such that no charge leaks out of the sphere. The energy difference of −6 Ry is taken between the core and valence band. The RMT × *K*_max_ cutoff is chosen to be 7. The IRelast package^28^ is used for the computation of elastic properties. For precise, reliable, and better results, 2000 *k*-points are taken for computation of all the properties.

## Results and discussions

### Structural characteristics

**Fig. 1:**
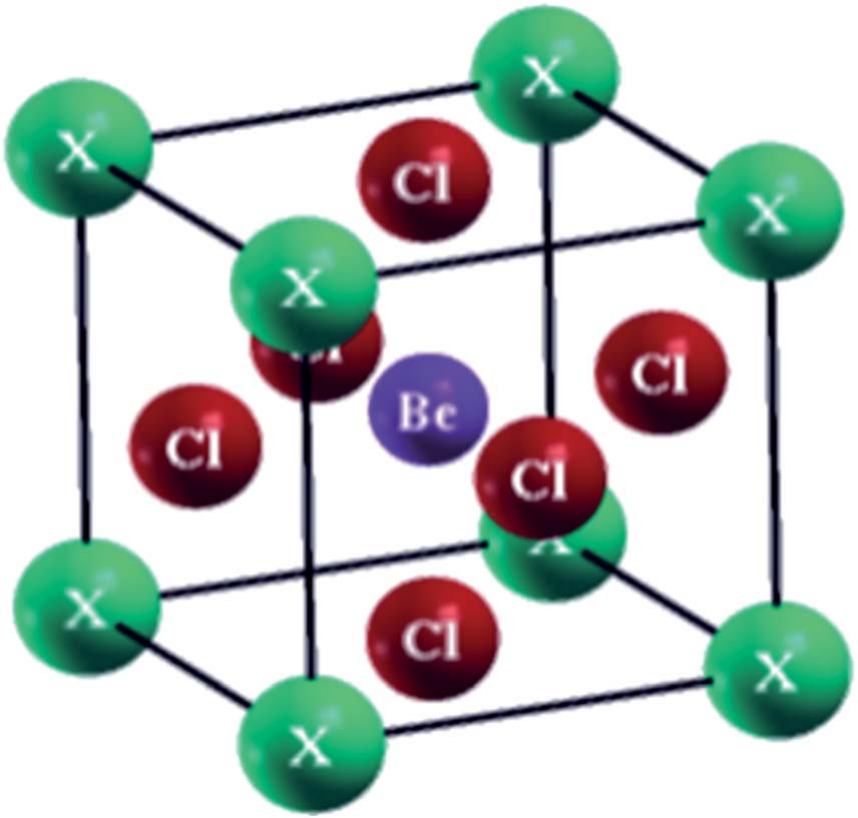
Crystal structure of XBeCl_3_ obtained with XCryDen.

**Fig. 2:**
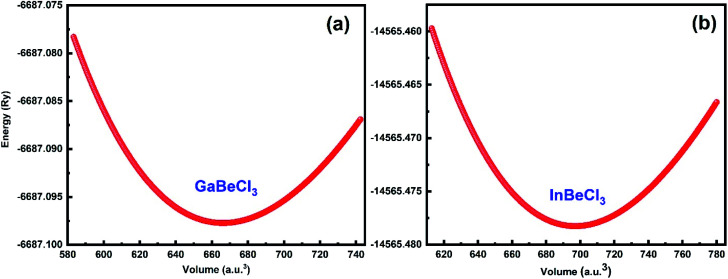
Total energy *vs.* volume of cubic perovskites XBeCl_3_.

The perovskites compounds XBeCl_3_ have an ideal cubic structure exhibiting the space group *pm̄*3*m* (#221). The occupied Wyckoff positions in the elementary cell are (0, 0, 0), (0.5, 0.5, 0.5) and (0, 0.5, 0.5) as depicted in Fig. 1. The volume *vs.* energy is fitted by the Birch–Murnaghan equation of state (EOS) as shown in Fig. 2.^29^ From this fit, the determined ground state characteristics *i*–*e* the lattice constant (*a*_o_), bulk modulus (*B*), the minimum energy (*E*_o_), and its pressure derivative (*B*′) are given in the table. No previous experimental or theoretical results are available for the GaBeCl_3_ and InBeCl_3_ compounds for comparison with the current calculations (Table 1).

**Table 1:** Calculated *a*_o_ (*Å*), *B* (GPa), *E*_o_ (Ry), *B*′ (GPa) for cubic perovskites GaBeCl_3_ and InBeCl_3_

Compounds	*a*_o_	*B*	*B*′	*E*_o_
GaBeCl_3_	4.621	65.578	5.371	−6687.0977
InBeCl_3_	4.586	193.255	42.380	−14565.4784

## Elastic characteristics

The elastic constants are basic and necessary parameters used to describe the mechanical properties of materials.^30^ For cubic systems, there are three completely independent elastic constants *i*–*e C*_11_, *C*_12_, and *C*_44_.^31^ Using these elastic constants, the other parameters *e.g. G*, *ν*, *A*, *E*, and *B*/*G* can be calculated using Voigt–Reuss–Hill eqn (1)–(6).^32–34^1
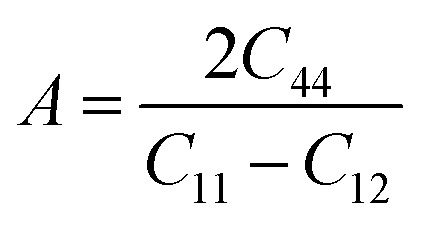
2
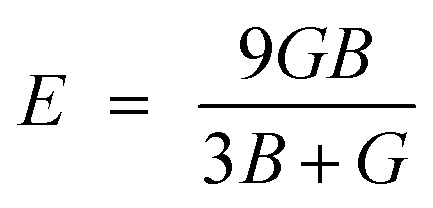
3
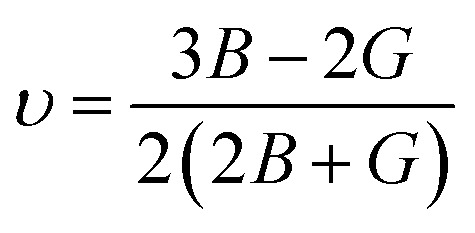
4
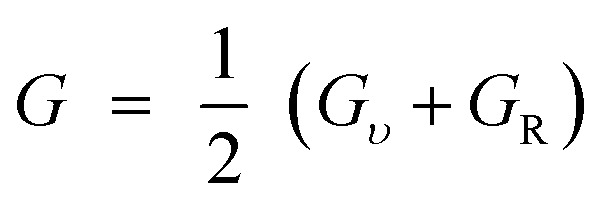
5
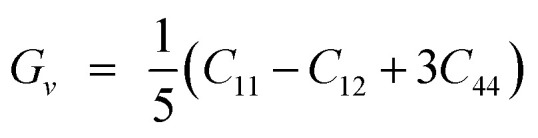
6
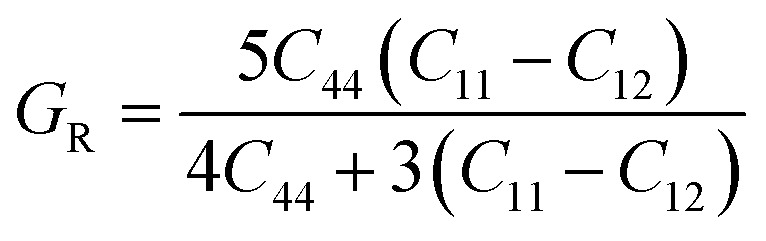


The “*B*” and “*G*” determine the hardness of the materials.^35^ Similarly, the material will be brittle, if *υ* <0.26 and *B*/*G* <1.75 otherwise it will exhibit a ductile nature.^36^ Here both the compounds show the ductile nature. Furthermore, as for isotropic nature, the value of “*A*” should be equal to 1, if not then the material will have anisotropic nature. Therefore, GaBeCl_3_ and InBeCl_3_ compounds show anisotropic properties. All the obtained results are summarized in Table 2.

**Table 2:** Calculated *C*_11_(GPa), *C*_12_(GPa), *C*_44_(GPa), *G*(GPa), *ν*, *A*, *E*(GPa), and *B*/*G* for GaBeCl_3_ compounds

Compounds	*C*_11_	*C*_12_	*C*_44_	*A*	*G*	*E*	*v*	*B*/*G*
GaBeCl_3_	94.9636	48.1999	52.4496	2.2432	38.6754	96.9643	0.3515	1.6956
InBeCl_3_	332.7265	107.3298	161.7959	1.4356	139.9710	338.2573	0.3582	1.3807

### Electronic characteristics

**Fig. 3:**
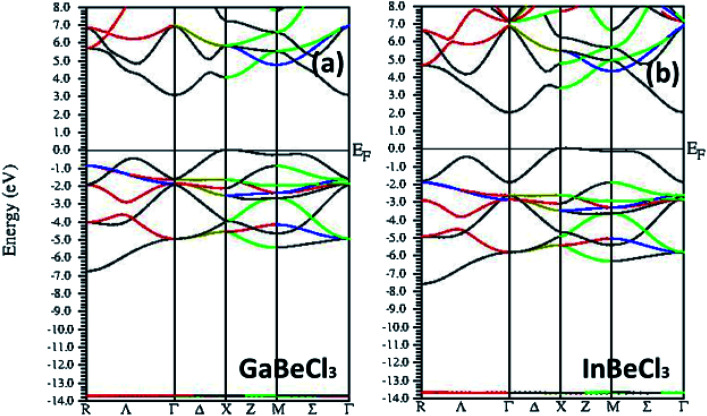
Band-structures of XBeCl_3_ along with the high symmetry directions. (a) GaBeCl_3_ (b) InBeCl_3_.

To study the electronic properties of the compounds, their energy band structures along the highly symmetrical points in the first Brillouin zone are calculated. Fig. 3(a) and (b) illustrates the band-structures of GaBeCl_3_ and InBeCl_3_ respectively. It can be observed that in both the compounds, the maximum of the valence band and minimum of the conduction band is located at *X* and *Γ* and the two bands do not overlap each other. Therefore, the material GaBeCl_3_ can be regarded as semiconducting which possessing an indirect band gap of ∼3.08 eV, while InBeCl_3_ possesses a band gap of 2.04 eV and thus a semiconductor.

**Fig. 4:**
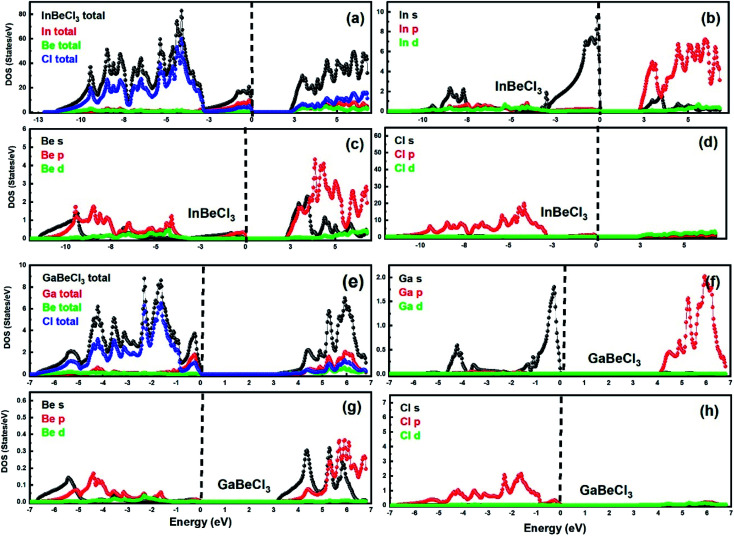
(a–d) TDOS and PDOS of InBeCl_3_. (e–h) TDOS and PDOS of GaBeCl_3_.

To better understand the electronic properties, the TDOS and PDOS are calculated for both compounds. Fig. 4(a–d) illustrates the TDOS and PDOS for the GaBeCl_3_ compound. TDOS is divided into three regions. In the first region from −7 to −1, Cl (p-states) contributes the maximum. In the second region from −1 to 0, the contribution of Ga (4p-states), as well as Cl (3p-states) atoms, is large. These are the regions below the Fermi energy (*E*_F_). Above *E*_F_, again the contribution of Ga (4p-states) and Cl (3p-states) atoms is large. Consequently, in both the valence band as well as the conduction band Ga and Cl, p orbital states are maximum. Similarly, Fig. 4(e–h) shows the TDOS and PDOS for the InBeCl_3_ compound. Here the contribution of Cl (3p-states) is large in the energy range of −12 to −3 eV. However, in the region from −3 to 0, In (5s-states) atoms contribution is large. In addition, a Cl (3p-states) atom also makes a certain contribution. Above *E*_F_, in the energy range from 3–8 eV, Cl (3p-states) atoms participation is maximum. Hence the contribution of states in the valence band of InBeCl_3_ is due to Cl (3p-states) and In (5s-states) and in that of the conduction band is due to the Cl (3p-states) only. The result of TDOS and PDOS confirms the band structure reports.

The electronic properties or the band gap varies with the change in *A*-site, *B*-site and *X*-site (halide) ion. As well as when the calculations method is changed. Several examples are listed in Table 3.

**Table 3:** Calculated bandgaps for some compounds

Compound	LDA	mBJ
CsCaF_3_ (ref. 37)	6.1	*
CsBaF_3_ (ref. 31)	5.02	8.78
LiBaF_3_	7.83	*
BiGaO_3_ (ref. 38)	*	2.46
BiInO_3_ (ref. 38)	*	1.05

## Optical properties

The response of the materials to the incident photons are calculated in the energy range 0–40 eV. The complex dielectric function (eqn (7)) can be separated into two parts *i*–*e* real (*ε*_1_(*ω*)) and imaginary (*ε*_2_(*ω*)) part.^39^

7 *ε*(*ω*) = *ε*_1_(*ω*) + *iε*_2_(*ω*)

**Fig. 5:**
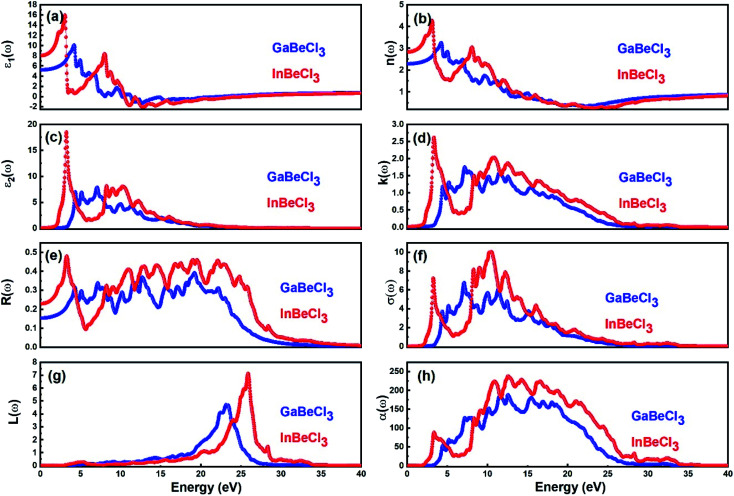
Calculated (a) *ε*_1_(*ω*), (b) *n*(*ω*), (c) *ε*_2_(*ω*) (d) *k*(*ω*) (e) *R*(*ω*) (f) *σ*(*ω*) (g) *L*(*ω*) and (h) *α*(*ω*) of XBeCl_3_ compounds.

Here *ε*_1_(*ω*) and *ε*_2_(*ω*) are related to the absorption and the band structure respectively.^40,41^Fig. 5(a and c) illustrates *ε*_1_(*ω*) and *ε*_2_(*ω*) spectrum respectively. From Fig. 5(a) it can be seen that the threshold energy or absorption edge^20^ for GaBeCl_3_ and InBeCl_3_ is located at ∼7.178 eV and ∼3.27 eV respectively. Similarly, the static dielectric constant *ε*_1_(0) (5(c)) for GaBeCl_3_ and InBeCl_3_ is situated at 5.1 and 8.08, while the maximum peaks are found at 4.37 and 3.10 after this a decrease can be seen in both the plots, following another maximum.

Fig. 5(b) shows *n*(*ω*) for both the compounds the static refractive index *n*(0) is found to be 2.32 and 2.88 for GaBeCl_3_ and InBeCl_3_ respectively. The value of *n*(*ω*) increases and reaches a maximum value of 3.27 at 4.40 and 4.26 at 3.1 eV. This lies in the UV region of the light spectrum. Furthermore, it is observed that behavior of *n*(*ω*) and *k*(*ω*) is similar to *ε*_1_(*ω*) and *ε*_2_(*ω*) for both the compounds.

Fig. 5(e) displays the *R*(*ω*) spectra for both the compounds. The results depict the maximum reflectivity of 38% and 48% at 19.24 eV and 3.3 eV for GaBeCl_3_ and InBeCl_3_ respectively. Similarly, Fig. 5(f–h) depicts (*σ*(*ω*), *L*(*ω*)) and *α*(*ω*) for GaBeCl_3_ and InBeCl_3_ compounds. The highest conductivity *i*–*e* 6.71 Ω^−1^ cm^−1^ is obtained at 7.04 eV and 9.98 Ω^−1^ cm^−1^ at 10.50 eV for GaBeCl_3_ and InBeCl_3_ respectively. The resonant energy losses are observed at 23.25 eV and 25.97 eV for both the compounds respectively. In addition, the point at which the material starts absorption is found to be at 2.23 eV and 3.23 eV for GaBeCl_3_ and InBeCl_3_ compounds respectively.

## Conclusion

In summary, the TB-mBJ approximation based on DFT is used to investigate the structural, elastic, electronic, and optical properties of ABeCl_3_ (A = Ga, In). It is reported that with the change in cation from Ga to In, the lattice constant and ground state energy decreases while bulk modulus and pressure derivative of bulk modulus increase. The elastic properties confirmed the anisotropic and ductile nature of both the compounds. Furthermore, the studied compounds have mechanical stability which is the key requirement for applications in high-performance electronic devices. The ionic type of chemical bonding is present in the compounds. In addition, the investigated electronic-band structure reveals the narrow band-gap semiconducting nature of the compounds with the indirect gap of ∼3.1 eV along (*X*–*Γ*) for GaBeCl_3_ and InBeCl_3_compounds. The results of TDOS and PDOS for GaBeCl_3_ and InBeCl_3_ compound shows that in both the valence band as well as the conduction band Ga (4p-states) and Cl (3p-states) atoms contribution are maximum for GaBeCl_3_. However, in InBeCl_3_ the contribution of Cl (3p-states) and In (5s-states) in the conduction band and that of Cl (3p-states) in the valence band is large. In addition, the optical properties of GaBeCl_3_ and InBeCl_3_ are calculated in the photon energy range of 0–40 eV. It is reported that *n*(*ω*) and *k*(*ω*) exhibit the same characteristics as *ε*_1_(*ω*) and *ε*_2_(*ω*) respectively. The computed properties of this work must be considered to understand and utilize the possible technical benefits in device manufacturing in semiconducting industries. Optical properties are of great significance in radiation detection and laser technology.

## Conflicts of interest

There are no conflicts of interest.

## Supplementary Material
