# Correction: Insight into the exemplary structural, elastic, electronic and optical nature of GaBeCl_3_ and InBeCl_3_: a DFT study

**DOI:** 10.1039/d2ra90025d

**Published:** 2022-03-24

**Authors:** Saima Ahmad Shah, Mudasser Husain, Nasir Rahman, Mohammad Sohail, Rajwali Khan, Abid Ali Khan, Asad Ullah, Shaimaa A. M. Abdelmohsen, Ashraf M. M. Abdelbacki, Ahmed M. El-Sabrout, Hosam O. Elansary, Aurangzeb Khan

**Affiliations:** Department of Physics, Shaheed Benazir Bhutto Women University Peshawar Pakistan; Department of Physics, Abdul Wali Khan University Mardan Pakistan akhan@awkum.edu.pk; Department of Physics, University of Lakki Marwat 28420 Lakki Marwat Khyber Pakhtunkhwa Pakistan nasir@ulm.edu.pk; Department of Chemical Sciences, University of Lakki Marwat 28420 Lakki Marwat Khyber Pakhtunkhwa Pakistan; Department of Mathematical Sciences, University of Lakki Marwat 28420 Lakki Marwat Khyber Pakhtunkhwa Pakistan; Department of Physics, College of Science, Princess Nourah Bint Abdulrahman University P. O. Box 84428 Riyadh 11681 Saudi Arabia; Deanship of Skills Development, King Saud University Riyadh 11451 Saudi Arabia; Department of Applied Entomology and Zoology, Faculty of Agriculture (EL-Shatby), Alexandria University Alexandria 21545 Egypt; Plant Production Department, College of Food and Agriculture Sciences, King Saud University Riyadh 11451 Saudi Arabia; University of Lakki Marwat 28420 Lakki Marwat Khyber Pakhtunkhwa Pakistan

## Abstract

Correction for ‘Insight into the exemplary structural, elastic, electronic and optical nature of GaBeCl_3_ and InBeCl_3_: a DFT study’ by Saima Ahmad Shah *et al.*, *RSC Adv.*, 2022, **12**, 8172–8177, DOI: 10.1039/D2RA00943A.

The authors regret that three of the authors were associated with the incorrect affiliations. The correct list of authors and affiliations is as shown here.

The Royal Society of Chemistry apologises for these errors and any consequent inconvenience to authors and readers.

## Supplementary Material

